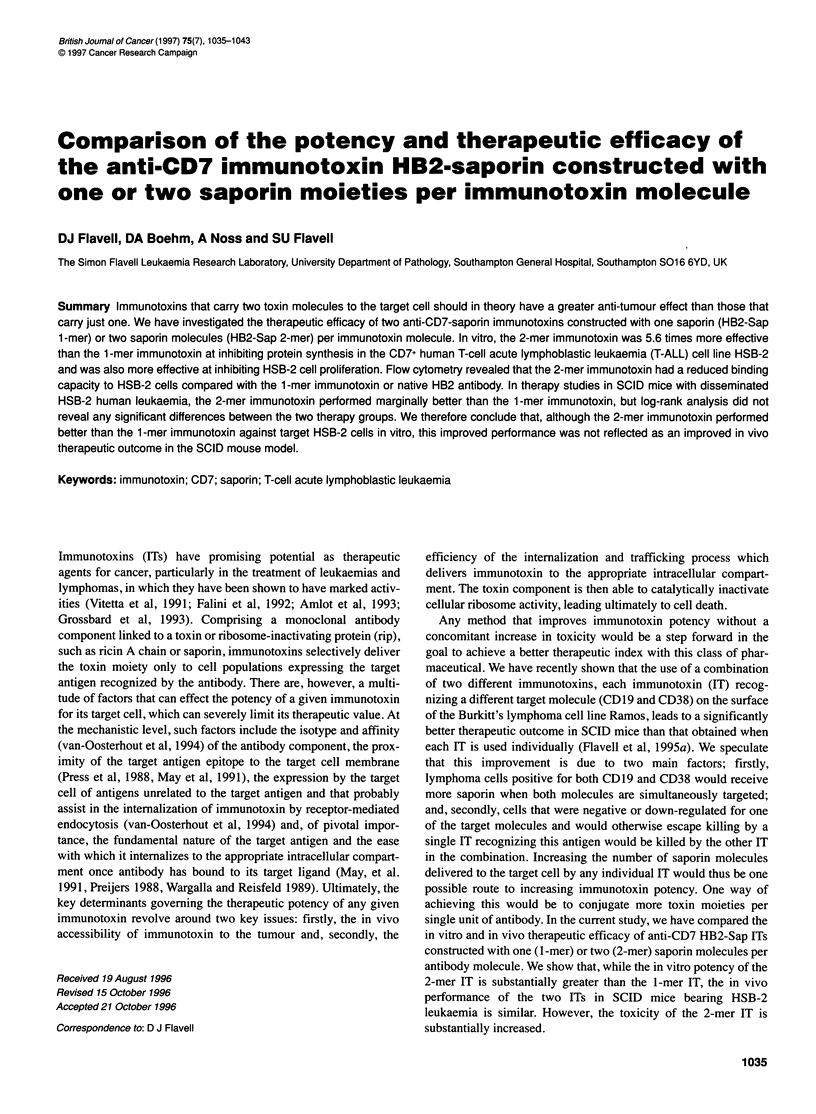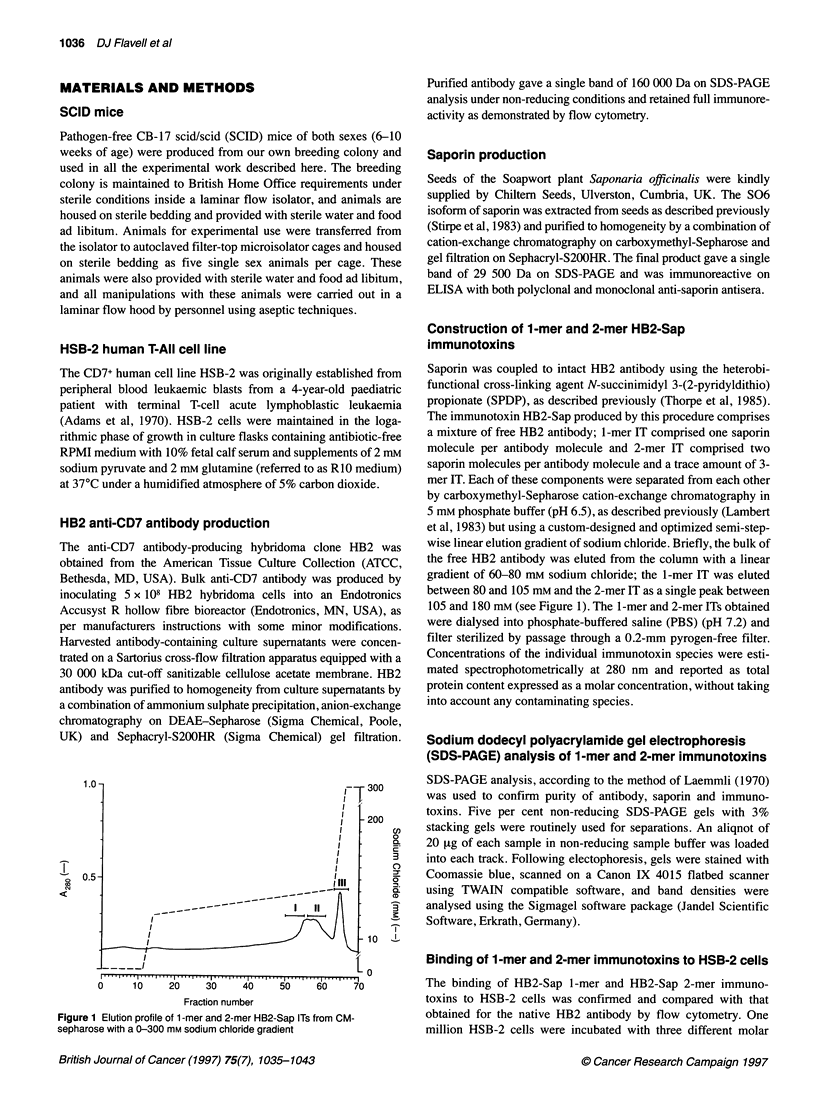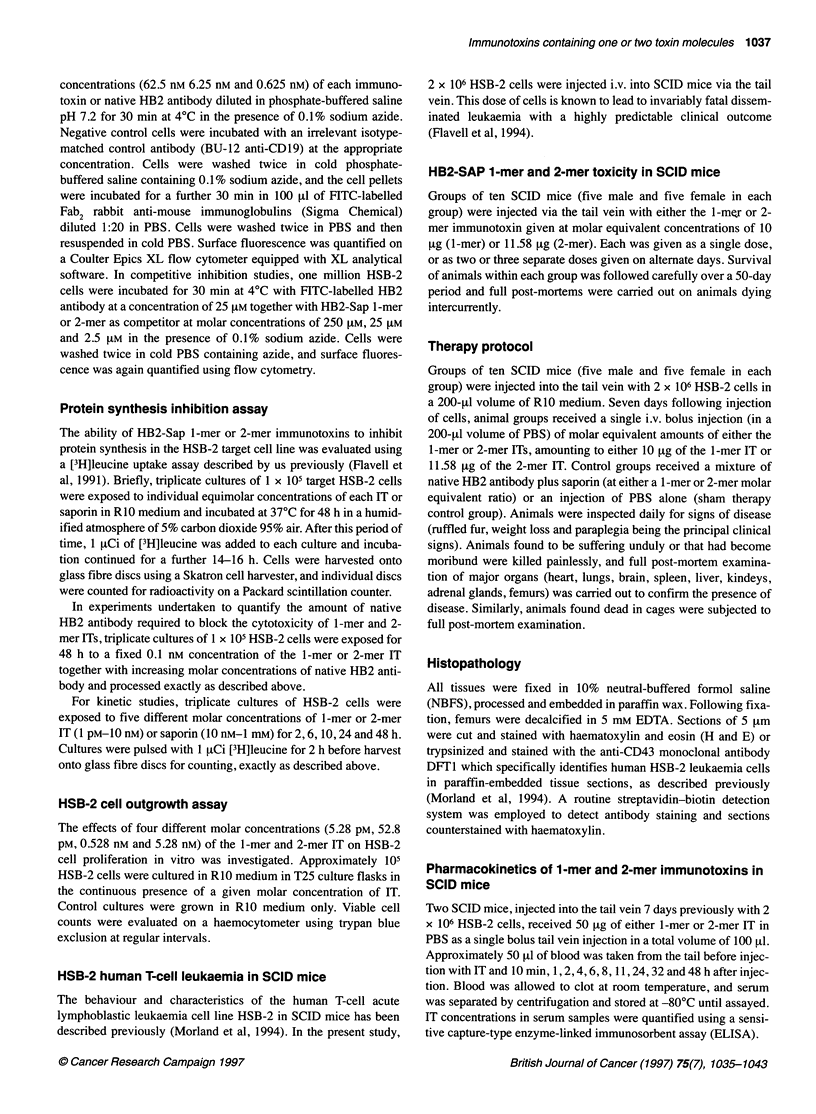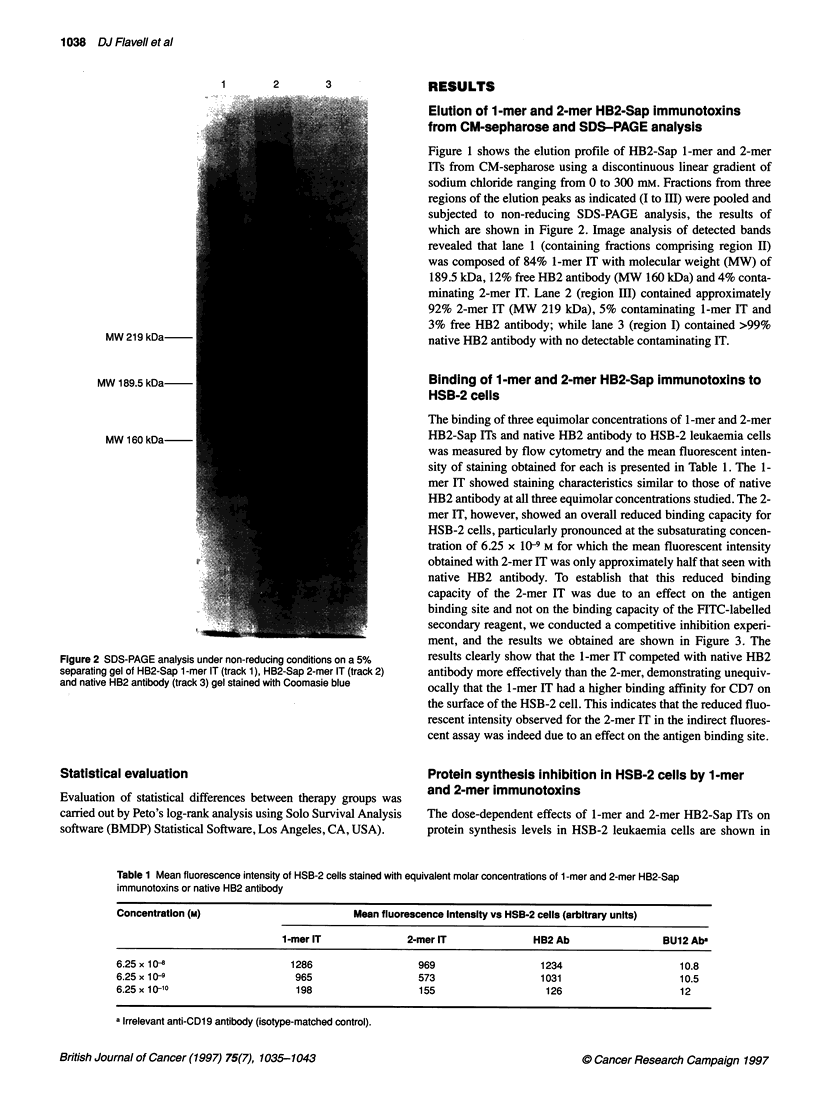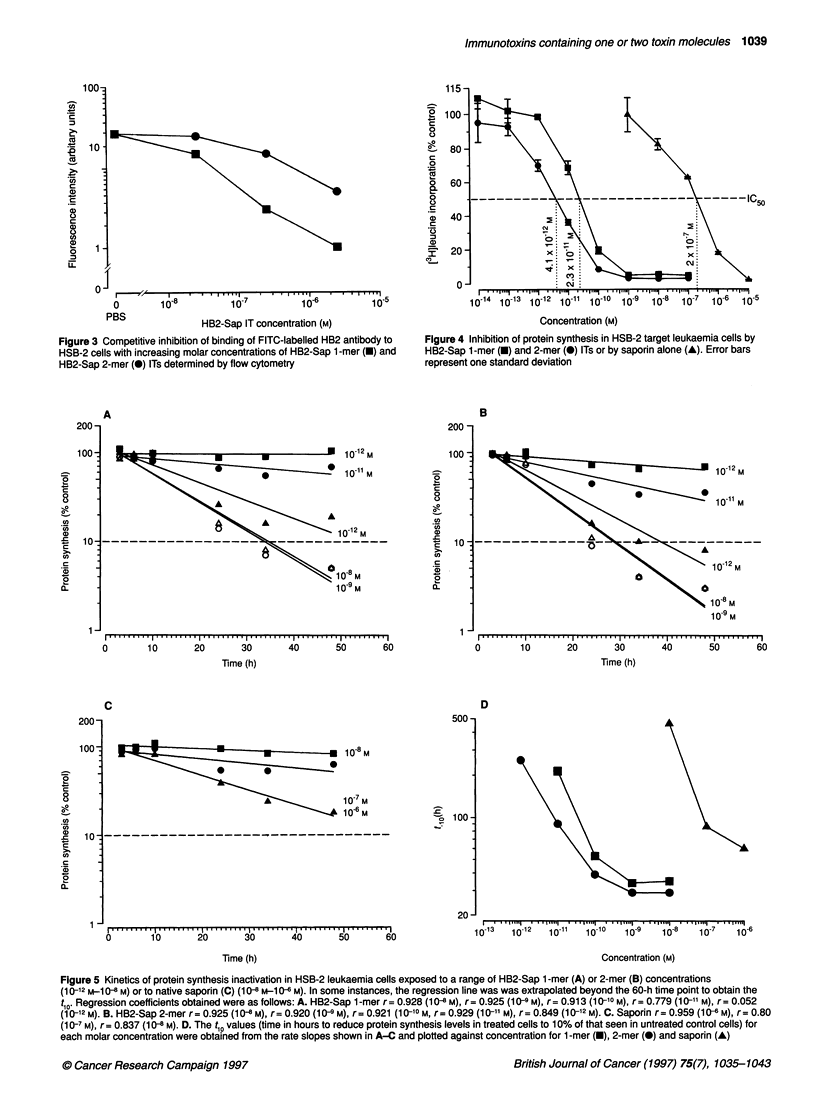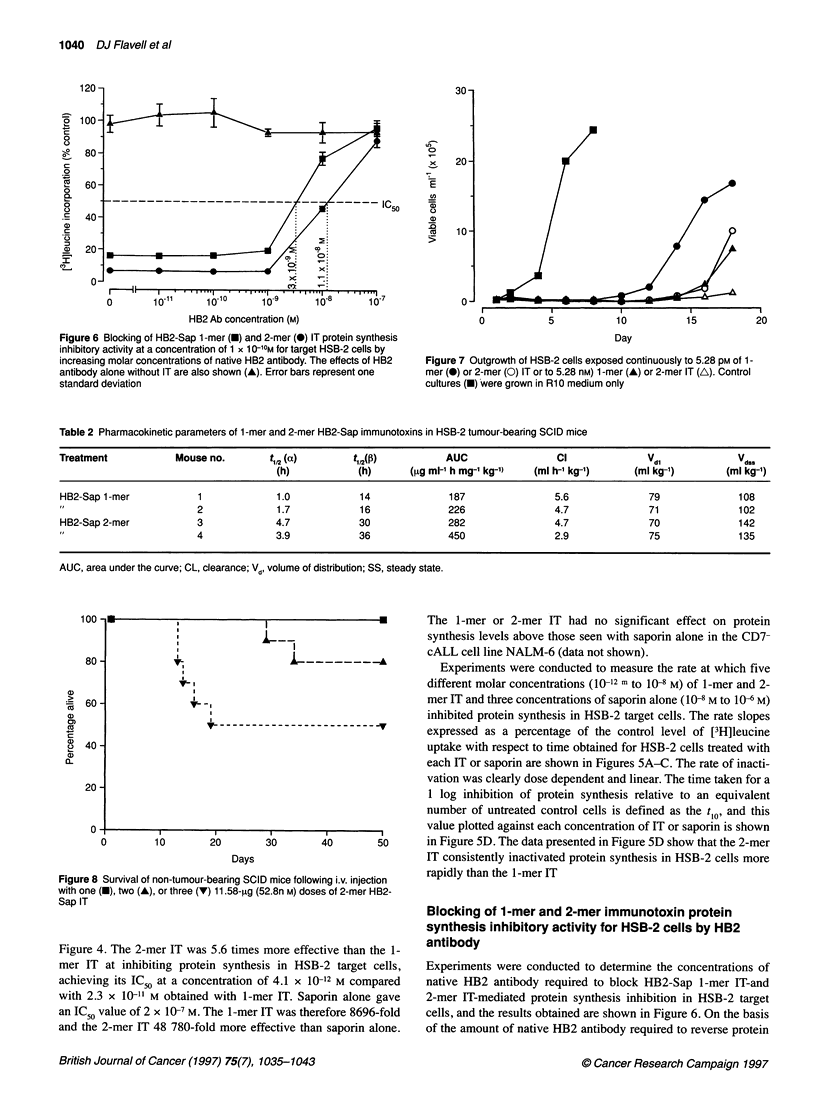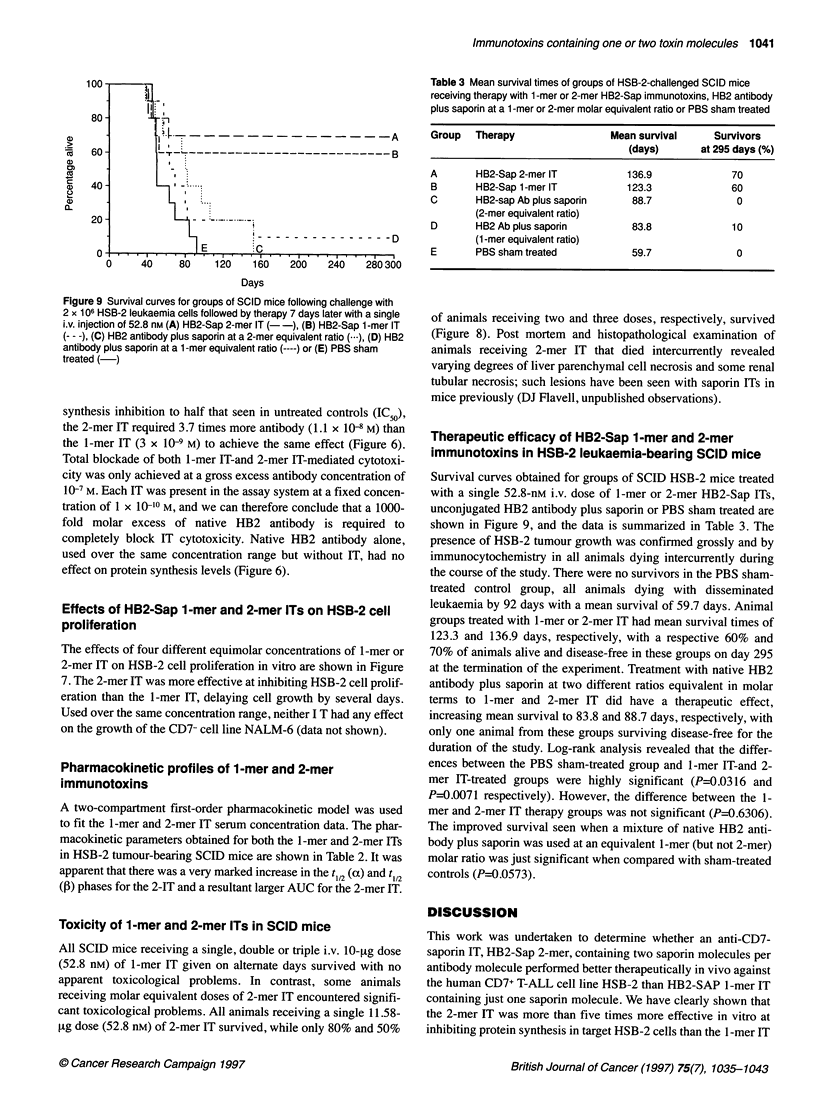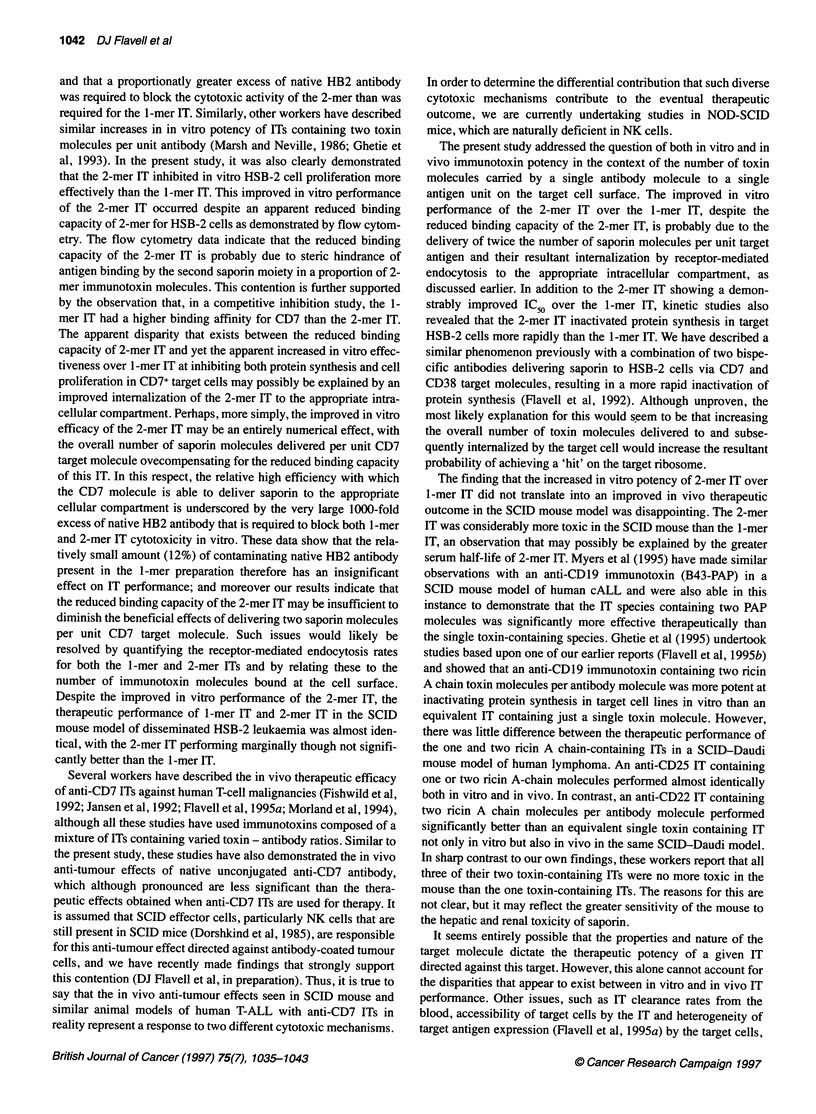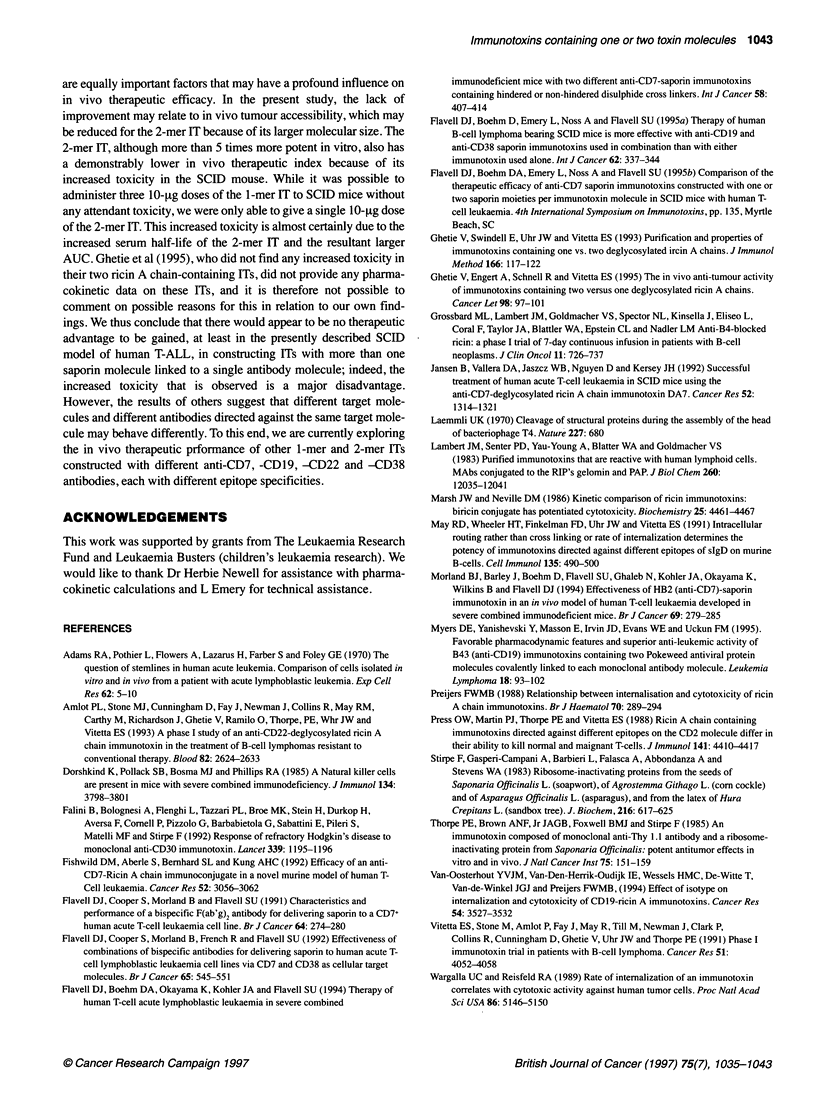# Comparison of the potency and therapeutic efficacy of the anti-CD7 immunotoxin HB2-saporin constructed with one or two saporin moieties per immunotoxin molecule.

**DOI:** 10.1038/bjc.1997.177

**Published:** 1997

**Authors:** D. J. Flavell, D. A. Boehm, A. Noss, S. U. Flavell

**Affiliations:** The Simon Flavell Leukaemia Research Laboratory, University Department of Pathology, Southampton General Hospital, UK.

## Abstract

**Images:**


*British Journal of Cancer* (1997) **75**(7), 1035–1043
© 1997 Cancer Research Campaign

### Comparison of the potency and therapeutic efficacy of the anti-CD7 immunotoxin HB2-saporin constructed with one or two saporin moieties per immunotoxin molecule

**DJ Flavell, DA Boehm, A Noss and SU Flavell**

The Simon Flavell Leukaemia Research Laboratory, University Department of Pathology, Southampton General Hospital, Southampton SO16 6YD, UK

**Summary** Immunotoxins that carry two toxin molecules to the target cell should in theory have a greater anti-tumour effect than those that carry just one. We have investigated the therapeutic efficacy of two anti-CD7-saporin immunotoxins constructed with one saporin (HB2-Sap 1-mer) or two saporin molecules (HB2-Sap 2-mer) per immunotoxin molecule. In vitro, the 2-mer immunotoxin was 5.6 times more effective than the 1-mer immunotoxin at inhibiting protein synthesis in the CD7+ human T-cell acute lymphoblastic leukaemia (T-ALL) cell line HSB-2 and was also more effective at inhibiting HSB-2 cell proliferation. Flow cytometry revealed that the 2-mer immunotoxin had a reduced binding capacity to HSB-2 cells compared with the 1-mer immunotoxin or native HB2 antibody. In therapy studies in SCID mice with disseminated HSB-2 human leukaemia, the 2-mer immunotoxin performed marginally better than the 1-mer immunotoxin, but log-rank analysis did not reveal any significant differences between the two therapy groups. We therefore conclude that, although the 2-mer immunotoxin performed better than the 1-mer immunotoxin against target HSB-2 cells in vitro, this improved performance was not reflected as an improved in vivo therapeutic outcome in the SCID mouse model.

**Keywords:** immunotoxin; CD7; saporin; T-cell acute lymphoblastic leukaemia

Immunotoxins (ITs) have promising potential as therapeutic agents for cancer, particularly in the treatment of leukaemias and lymphomas, in which they have been shown to have marked activities (Vitetta et al, 1991; Falini et al, 1992; Amlot et al, 1993; Grossbard et al, 1993). Comprising a monoclonal antibody component linked to a toxin or ribosome-inactivating protein (rip), such as ricin A chain or saporin, immunotoxins selectively deliver the toxin moiety only to cell populations expressing the target antigen recognized by the antibody. There are, however, a multitude of factors that can effect the potency of a given immunotoxin for its target cell, which can severely limit its therapeutic value. At the mechanistic level, such factors include the isotype and affinity (van-Oosterhout et al, 1994) of the antibody component, the proximity of the target antigen epitope to the target cell membrane (Press et al, 1988, May et al, 1991), the expression by the target cell of antigens unrelated to the target antigen and that probably assist in the internalization of immunotoxin by receptor-mediated endocytosis (van-Oosterhout et al, 1994) and, of pivotal importance, the fundamental nature of the target antigen and the ease with which it internalizes to the appropriate intracellular compartment once antibody has bound to its target ligand (May, et al. 1991, Preijers 1988, Wargalla and Reisfeld 1989). Ultimately, the key determinants governing the therapeutic potency of any given immunotoxin revolve around two key issues: firstly, the in vivo accessibility of immunotoxin to the tumour and, secondly, the efficiency of the internalization and trafficking process which delivers immunotoxin to the appropriate intracellular compartment. The toxin component is then able to catalytically inactivate cellular ribosome activity, leading ultimately to cell death.

*Received 19 August 1996*
*Revised 15 October 1996*
*Accepted 21 October 1996*

*Correspondence to*: D J Flavell

Any method that improves immunotoxin potency without a concomitant increase in toxicity would be a step forward in the goal to achieve a better therapeutic index with this class of pharmaceutical. We have recently shown that the use of a combination of two different immunotoxins, each immunotoxin (IT) recognizing a different target molecule (CD19 and CD38) on the surface of the Burkitt's lymphoma cell line Ramos, leads to a significantly better therapeutic outcome in SCID mice than that obtained when each IT is used individually (Flavell et al, 1995*a*). We speculate that this improvement is due to two main factors; firstly, lymphoma cells positive for both CD19 and CD38 would receive more saporin when both molecules are simultaneously targeted; and, secondly, cells that were negative or down-regulated for one of the target molecules and would otherwise escape killing by a single IT recognizing this antigen would be killed by the other IT in the combination. Increasing the number of saporin molecules delivered to the target cell by any individual IT would thus be one possible route to increasing immunotoxin potency. One way of achieving this would be to conjugate more toxin moieties per single unit of antibody. In the current study, we have compared the in vitro and in vivo therapeutic efficacy of anti-CD7 HB2-Sap ITs constructed with one (1-mer) or two (2-mer) saporin molecules per antibody molecule. We show that, while the in vitro potency of the 2-mer IT is substantially greater than the 1-mer IT, the in vivo performance of the two ITs in SCID mice bearing HSB-2 leukaemia is similar. However, the toxicity of the 2-mer IT is substantially increased.

### MATERIALS AND METHODS

### SCID mice

Pathogen-free CB-17 scid/scid (SCID) mice of both sexes (6–10 weeks of age) were produced from our own breeding colony and used in all the experimental work described here. The breeding colony is maintained to British Home Office requirements under sterile conditions inside a laminar flow isolator, and animals are housed on sterile bedding and provided with sterile water and food ad libitum. Animals for experimental use were transferred from the isolator to autoclaved filter-top microisolator cages and housed on sterile bedding as five single sex animals per cage. These animals were also provided with sterile water and food ad libitum, and all manipulations with these animals were carried out in a laminar flow hood by personnel using aseptic techniques.

### HSB-2 human T-All cell line

The CD7+ human cell line HSB-2 was originally established from peripheral blood leukaemic blasts from a 4-year-old paediatric patient with terminal T-cell acute lymphoblastic leukaemia (Adams et al, 1970). HSB-2 cells were maintained in the logarithmic phase of growth in culture flasks containing antibiotic-free RPMI medium with 10% fetal calf serum and supplements of 2 mM sodium pyruvate and 2 mM glutamine (referred to as R10 medium) at 37°C under a humidified atmosphere of 5% carbon dioxide.

### HB2 anti-CD7 antibody production

The anti-CD7 antibody-producing hybridoma clone HB2 was obtained from the American Tissue Culture Collection (ATCC, Bethesda, MD, USA). Bulk anti-CD7 antibody was produced by inoculating $5 \times 10^8$ HB2 hybridoma cells into an Endotronics Accusyst R hollow fibre bioreactor (Endotronics, MN, USA), as per manufacturers instructions with some minor modifications. Harvested antibody-containing culture supernatants were concentrated on a Sartorius cross-flow filtration apparatus equipped with a 30 000 kDa cut-off sanitizable cellulose acetate membrane. HB2 antibody was purified to homogeneity from culture supernatants by a combination of ammonium sulphate precipitation, anion-exchange chromatography on DEAE–Sepharose (Sigma Chemical, Poole, UK) and Sephacryl-S200HR (Sigma Chemical) gel filtration.

Figure 1 Elution profile of 1-mer and 2-mer HB2-Sap ITs from CM-sepharose with a 0–300 mM sodium chloride gradient

Purified antibody gave a single band of 160 000 Da on SDS-PAGE analysis under non-reducing conditions and retained full immunoreactivity as demonstrated by flow cytometry.

### Saporin production

Seeds of the Soapwort plant *Saponaria officinalis* were kindly supplied by Chiltern Seeds, Ulverston, Cumbria, UK. The SO6 isoform of saporin was extracted from seeds as described previously (Stirpe et al, 1983) and purified to homogeneity by a combination of cation-exchange chromatography on carboxymethyl-Sepharose and gel filtration on Sephacryl-S200HR. The final product gave a single band of 29 500 Da on SDS-PAGE and was immunoreactive on ELISA with both polyclonal and monoclonal anti-saporin antisera.

### Construction of 1-mer and 2-mer HB2-Sap immunotoxins

Saporin was coupled to intact HB2 antibody using the heterobifunctional cross-linking agent *N*-succinimidyl 3-(2-pyridyldithio) propionate (SPDP), as described previously (Thorpe et al, 1985). The immunotoxin HB2-Sap produced by this procedure comprises a mixture of free HB2 antibody; 1-mer IT comprised one saporin molecule per antibody molecule and 2-mer IT comprised two saporin molecules per antibody molecule and a trace amount of 3-mer IT. Each of these components were separated from each other by carboxymethyl-Sepharose cation-exchange chromatography in 5 mM phosphate buffer (pH 6.5), as described previously (Lambert et al, 1983) but using a custom-designed and optimized semi-stepwise linear elution gradient of sodium chloride. Briefly, the bulk of the free HB2 antibody was eluted from the column with a linear gradient of 60–80 mM sodium chloride; the 1-mer IT was eluted between 80 and 105 mM and the 2-mer IT as a single peak between 105 and 180 mM (see Figure 1). The 1-mer and 2-mer ITs obtained were dialysed into phosphate-buffered saline (PBS) (pH 7.2) and filter sterilized by passage through a 0.2-mm pyrogen-free filter. Concentrations of the individual immunotoxin species were estimated spectrophotometrically at 280 nm and reported as total protein content expressed as a molar concentration, without taking into account any contaminating species.

### Sodium dodecyl polyacrylamide gel electrophoresis (SDS-PAGE) analysis of 1-mer and 2-mer immunotoxins

SDS-PAGE analysis, according to the method of Laemmli (1970) was used to confirm purity of antibody, saporin and immunotoxins. Five per cent non-reducing SDS-PAGE gels with 3% stacking gels were routinely used for separations. An aliqnot of 20 μg of each sample in non-reducing sample buffer was loaded into each track. Following electophoresis, gels were stained with Coomassie blue, scanned on a Canon IX 4015 flatbed scanner using TWAIN compatible software, and band densities were analysed using the Sigmagel software package (Jandel Scientific Software, Erkrath, Germany).

### Binding of 1-mer and 2-mer immunotoxins to HSB-2 cells

The binding of HB2-Sap 1-mer and HB2-Sap 2-mer immunotoxins to HSB-2 cells was confirmed and compared with that obtained for the native HB2 antibody by flow cytometry. One million HSB-2 cells were incubated with three different molar

 © Cancer Research Campaign 1997

concentrations (62.5 nM 6.25 nM and 0.625 nM) of each immunotoxin or native HB2 antibody diluted in phosphate-buffered saline pH 7.2 for 30 min at 4°C in the presence of 0.1% sodium azide. Negative control cells were incubated with an irrelevant isotype-matched control antibody (BU-12 anti-CD19) at the appropriate concentration. Cells were washed twice in cold phosphate-buffered saline containing 0.1% sodium azide, and the cell pellets were incubated for a further 30 min in 100 μl of FITC-labelled $Fab_2$ rabbit anti-mouse immunoglobulins (Sigma Chemical) diluted 1:20 in PBS. Cells were washed twice in PBS and then resuspended in cold PBS. Surface fluorescence was quantified on a Coulter Epics XL flow cytometer equipped with XL analytical software. In competitive inhibition studies, one million HSB-2 cells were incubated for 30 min at 4°C with FITC-labelled HB2 antibody at a concentration of 25 μM together with HB2-Sap 1-mer or 2-mer as competitor at molar concentrations of 250 μM, 25 μM and 2.5 μM in the presence of 0.1% sodium azide. Cells were washed twice in cold PBS containing azide, and surface fluorescence was again quantified using flow cytometry.

### Protein synthesis inhibition assay

The ability of HB2-Sap 1-mer or 2-mer immunotoxins to inhibit protein synthesis in the HSB-2 target cell line was evaluated using a [$^3$H]leucine uptake assay described by us previously (Flavell et al, 1991). Briefly, triplicate cultures of $1 \times 10^5$ target HSB-2 cells were exposed to individual equimolar concentrations of each IT or saporin in R10 medium and incubated at 37°C for 48 h in a humidified atmosphere of 5% carbon dioxide 95% air. After this period of time, 1 μCi of [$^3$H]leucine was added to each culture and incubation continued for a further 14–16 h. Cells were harvested onto glass fibre discs using a Skatron cell harvester, and individual discs were counted for radioactivity on a Packard scintillation counter.

In experiments undertaken to quantify the amount of native HB2 antibody required to block the cytotoxicity of 1-mer and 2-mer ITs, triplicate cultures of $1 \times 10^5$ HSB-2 cells were exposed for 48 h to a fixed 0.1 nM concentration of the 1-mer or 2-mer IT together with increasing molar concentrations of native HB2 antibody and processed exactly as described above.

For kinetic studies, triplicate cultures of HSB-2 cells were exposed to five different molar concentrations of 1-mer or 2-mer IT (1 pM–10 nM) or saporin (10 nM–1 mM) for 2, 6, 10, 24 and 48 h. Cultures were pulsed with 1 μCi [$^3$H]leucine for 2 h before harvest onto glass fibre discs for counting, exactly as described above.

### HSB-2 cell outgrowth assay

The effects of four different molar concentrations (5.28 pM, 52.8 pM, 0.528 nM and 5.28 nM) of the 1-mer and 2-mer IT on HSB-2 cell proliferation in vitro was investigated. Approximately $10^5$ HSB-2 cells were cultured in R10 medium in T25 culture flasks in the continuous presence of a given molar concentration of IT. Control cultures were grown in R10 medium only. Viable cell counts were evaluated on a haemocytometer using trypan blue exclusion at regular intervals.

### HSB-2 human T-cell leukaemia in SCID mice

The behaviour and characteristics of the human T-cell acute lymphoblastic leukaemia cell line HSB-2 in SCID mice has been described previously (Morland et al, 1994). In the present study, $2 \times 10^6$ HSB-2 cells were injected i.v. into SCID mice via the tail vein. This dose of cells is known to lead to invariably fatal disseminated leukaemia with a highly predictable clinical outcome (Flavell et al, 1994).

### HB2-SAP 1-mer and 2-mer toxicity in SCID mice

Groups of ten SCID mice (five male and five female in each group) were injected via the tail vein with either the 1-mer or 2-mer immunotoxin given at molar equivalent concentrations of 10 μg (1-mer) or 11.58 μg (2-mer). Each was given as a single dose, or as two or three separate doses given on alternate days. Survival of animals within each group was followed carefully over a 50-day period and full post-mortems were carried out on animals dying intercurrently.

### Therapy protocol

Groups of ten SCID mice (five male and five female in each group) were injected into the tail vein with $2 \times 10^6$ HSB-2 cells in a 200-μl volume of R10 medium. Seven days following injection of cells, animal groups received a single i.v. bolus injection (in a 200-μl volume of PBS) of molar equivalent amounts of either the 1-mer or 2-mer ITs, amounting to either 10 μg of the 1-mer IT or 11.58 μg of the 2-mer IT. Control groups received a mixture of native HB2 antibody plus saporin (at either a 1-mer or 2-mer molar equivalent ratio) or an injection of PBS alone (sham therapy control group). Animals were inspected daily for signs of disease (ruffled fur, weight loss and paraplegia being the principal clinical signs). Animals found to be suffering unduly or that had become moribund were killed painlessly, and full post-mortem examination of major organs (heart, lungs, brain, spleen, liver, kindeys, adrenal glands, femurs) was carried out to confirm the presence of disease. Similarly, animals found dead in cages were subjected to full post-mortem examination.

### Histopathology

All tissues were fixed in 10% neutral-buffered formol saline (NBFS), processed and embedded in paraffin wax. Following fixation, femurs were decalcified in 5 mM EDTA. Sections of 5 μm were cut and stained with haematoxylin and eosin (H and E) or trypsinized and stained with the anti-CD43 monoclonal antibody DFT1 which specifically identifies human HSB-2 leukaemia cells in paraffin-embedded tissue sections, as described previously (Morland et al, 1994). A routine streptavidin–biotin detection system was employed to detect antibody staining and sections counterstained with haematoxylin.

### Pharmacokinetics of 1-mer and 2-mer immunotoxins in SCID mice

Two SCID mice, injected into the tail vein 7 days previously with $2 \times 10^6$ HSB-2 cells, received 50 μg of either 1-mer or 2-mer IT in PBS as a single bolus tail vein injection in a total volume of 100 μl. Approximately 50 μl of blood was taken from the tail before injection with IT and 10 min, 1, 2, 4, 6, 8, 11, 24, 32 and 48 h after injection. Blood was allowed to clot at room temperature, and serum was separated by centrifugation and stored at –80°C until assayed. IT concentrations in serum samples were quantified using a sensitive capture-type enzyme-linked immunosorbent assay (ELISA).

© Cancer Research Campaign 1997

**Figure 2** SDS-PAGE analysis under non-reducing conditions on a 5% separating gel of HB2-Sap 1-mer IT (track 1), HB2-Sap 2-mer IT (track 2) and native HB2 antibody (track 3) gel stained with Coomasie blue

### Statistical evaluation

Evaluation of statistical differences between therapy groups was carried out by Peto's log-rank analysis using Solo Survival Analysis software (BMDP) Statistical Software, Los Angeles, CA, USA).

### RESULTS

### Elution of 1-mer and 2-mer HB2-Sap immunotoxins from CM-sepharose and SDS–PAGE analysis

Figure 1 shows the elution profile of HB2-Sap 1-mer and 2-mer ITs from CM-sepharose using a discontinuous linear gradient of sodium chloride ranging from 0 to 300 mM. Fractions from three regions of the elution peaks as indicated (I to III) were pooled and subjected to non-reducing SDS-PAGE analysis, the results of which are shown in Figure 2. Image analysis of detected bands revealed that lane 1 (containing fractions comprising region II) was composed of 84% 1-mer IT with molecular weight (MW) of 189.5 kDa, 12% free HB2 antibody (MW 160 kDa) and 4% contaminating 2-mer IT. Lane 2 (region III) contained approximately 92% 2-mer IT (MW 219 kDa), 5% contaminating 1-mer IT and 3% free HB2 antibody; while lane 3 (region I) contained >99% native HB2 antibody with no detectable contaminating IT.

### Binding of 1-mer and 2-mer HB2-Sap immunotoxins to HSB-2 cells

The binding of three equimolar concentrations of 1-mer and 2-mer HB2-Sap ITs and native HB2 antibody to HSB-2 leukaemia cells was measured by flow cytometry and the mean fluorescent intensity of staining obtained for each is presented in Table 1. The 1-mer IT showed staining characteristics similar to those of native HB2 antibody at all three equimolar concentrations studied. The 2-mer IT, however, showed an overall reduced binding capacity for HSB-2 cells, particularly pronounced at the subsaturating concentration of $6.25 \times 10^{-9}$ M for which the mean fluorescent intensity obtained with 2-mer IT was only approximately half that seen with native HB2 antibody. To establish that this reduced binding capacity of the 2-mer IT was due to an effect on the antigen binding site and not on the binding capacity of the FITC-labelled secondary reagent, we conducted a competitive inhibition experiment, and the results we obtained are shown in Figure 3. The results clearly show that the 1-mer IT competed with native HB2 antibody more effectively than the 2-mer, demonstrating unequivocally that the 1-mer IT had a higher binding affinity for CD7 on the surface of the HSB-2 cell. This indicates that the reduced fluorescent intensity observed for the 2-mer IT in the indirect fluorescent assay was indeed due to an effect on the antigen binding site.

### Protein synthesis inhibition in HSB-2 cells by 1-mer and 2-mer immunotoxins

The dose-dependent effects of 1-mer and 2-mer HB2-Sap ITs on protein synthesis levels in HSB-2 leukaemia cells are shown in

**Table 1** Mean fluorescence intensity of HSB-2 cells stained with equivalent molar concentrations of 1-mer and 2-mer HB2-Sap immunotoxins or native HB2 antibody

| Concentration (M) | Mean fluorescence intensity vs HSB-2 cells (arbitrary units) | | | |
|---|---|---|---|---|
| | 1-mer IT | 2-mer IT | HB2 Ab | BU12 Ab[a] |
| $6.25 \times 10^{-8}$ | 1286 | 969 | 1234 | 10.8 |
| $6.25 \times 10^{-9}$ | 965 | 573 | 1031 | 10.5 |
| $6.25 \times 10^{-10}$ | 198 | 155 | 126 | 12 |

[a] Irrelevant anti-CD19 antibody (isotype-matched control).

© Cancer Research Campaign 1997

**Figure 3** Competitive inhibition of binding of FITC-labelled HB2 antibody to HSB-2 cells with increasing molar concentrations of HB2-Sap 1-mer (■) and HB2-Sap 2-mer (●) ITs determined by flow cytometry

**Figure 4** Inhibition of protein synthesis in HSB-2 target leukaemia cells by HB2-Sap 1-mer (■) and 2-mer (●) ITs or by saporin alone (▲). Error bars represent one standard deviation

**Figure 5** Kinetics of protein synthesis inactivation in HSB-2 leukaemia cells exposed to a range of HB2-Sap 1-mer (**A**) or 2-mer (**B**) concentrations ($10^{-12}$ M–$10^{-8}$ M) or to native saporin (**C**) ($10^{-8}$ M–$10^{-6}$ M). In some instances, the regression line was was extrapolated beyond the 60-h time point to obtain the $t_{10}$. Regression coefficients obtained were as follows: **A.** HB2-Sap 1-mer $r$ = 0.928 ($10^{-8}$ M), $r$ = 0.925 ($10^{-9}$ M), $r$ = 0.913 ($10^{-10}$ M), $r$ = 0.779 ($10^{-11}$ M), $r$ = 0.052 ($10^{-12}$ M). **B.** HB2-Sap 2-mer $r$ = 0.925 ($10^{-8}$ M), $r$ = 0.920 ($10^{-9}$ M), $r$ = 0.921 ($10^{-10}$ M, $r$ = 0.929 ($10^{-11}$ M), $r$ = 0.849 ($10^{-12}$ M). **C.** Saporin $r$ = 0.959 ($10^{-6}$ M), $r$ = 0.80 ($10^{-7}$ M), $r$ = 0.837 ($10^{-8}$ M). **D.** The $t_{10}$ values (time in hours to reduce protein synthesis levels in treated cells to 10% of that seen in untreated control cells) for each molar concentration were obtained from the rate slopes shown in **A–C** and plotted against concentration for 1-mer (■), 2-mer (●) and saporin (▲)

**Figure 6** Blocking of HB2-Sap 1-mer (■) and 2-mer (●) IT protein synthesis inhibitory activity at a concentration of 1 × $10^{-10}$M for target HSB-2 cells by increasing molar concentrations of native HB2 antibody. The effects of HB2 antibody alone without IT are also shown (▲). Error bars represent one standard deviation

**Figure 7** Outgrowth of HSB-2 cells exposed continuously to 5.28 pM of 1-mer (●) or 2-mer (○) IT or to 5.28 nM) 1-mer (▲) or 2-mer IT (△). Control cultures (■) were grown in R10 medium only

**Table 2** Pharmacokinetic parameters of 1-mer and 2-mer HB2-Sap immunotoxins in HSB-2 tumour-bearing SCID mice

| Treatment | Mouse no. | $t_{1/2}$ (α) (h) | $t_{1/2}$(β) (h) | AUC (μg ml⁻¹ h mg⁻¹ kg⁻¹) | Cl (ml h⁻¹ kg⁻¹) | $V_{d1}$ (ml kg⁻¹) | $V_{dss}$ (ml kg⁻¹) |
|---|---|---|---|---|---|---|---|
| HB2-Sap 1-mer | 1 | 1.0 | 14 | 187 | 5.6 | 79 | 108 |
| " | 2 | 1.7 | 16 | 226 | 4.7 | 71 | 102 |
| HB2-Sap 2-mer | 3 | 4.7 | 30 | 282 | 4.7 | 70 | 142 |
| " | 4 | 3.9 | 36 | 450 | 2.9 | 75 | 135 |

AUC, area under the curve; CL, clearance; $V_d$, volume of distribution; SS, steady state.

**Figure 8** Survival of non-tumour-bearing SCID mice following i.v. injection with one (■), two (▲), or three (▼) 11.58-μg (52.8n M) doses of 2-mer HB2-Sap IT

Figure 4. The 2-mer IT was 5.6 times more effective than the 1-mer IT at inhibiting protein synthesis in HSB-2 target cells, achieving its $IC_{50}$ at a concentration of 4.1 × $10^{-12}$ M compared with 2.3 × $10^{-11}$ M obtained with 1-mer IT. Saporin alone gave an $IC_{50}$ value of 2 × $10^{-7}$ M. The 1-mer IT was therefore 8696-fold and the 2-mer IT 48 780-fold more effective than saporin alone.

The 1-mer or 2-mer IT had no significant effect on protein synthesis levels above those seen with saporin alone in the $CD7^-$ cALL cell line NALM-6 (data not shown).

Experiments were conducted to measure the rate at which five different molar concentrations ($10^{-12}$ m to $10^{-8}$ M) of 1-mer and 2-mer IT and three concentrations of saporin alone ($10^{-8}$ M to $10^{-6}$ M) inhibited protein synthesis in HSB-2 target cells. The rate slopes expressed as a percentage of the control level of [³H]leucine uptake with respect to time obtained for HSB-2 cells treated with each IT or saporin are shown in Figures 5A–C. The rate of inactivation was clearly dose dependent and linear. The time taken for a 1 log inhibition of protein synthesis relative to an equivalent number of untreated control cells is defined as the $t_{10}$, and this value plotted against each concentration of IT or saporin is shown in Figure 5D. The data presented in Figure 5D show that the 2-mer IT consistently inactivated protein synthesis in HSB-2 cells more rapidly than the 1-mer IT

### Blocking of 1-mer and 2-mer immunotoxin protein synthesis inhibitory activity for HSB-2 cells by HB2 antibody

Experiments were conducted to determine the concentrations of native HB2 antibody required to block HB2-Sap 1-mer IT-and 2-mer IT-mediated protein synthesis inhibition in HSB-2 target cells, and the results obtained are shown in Figure 6. On the basis of the amount of native HB2 antibody required to reverse protein

 © Cancer Research Campaign 1997

**Figure 9** Survival curves for groups of SCID mice following challenge with 2 × $10^6$ HSB-2 leukaemia cells followed by therapy 7 days later with a single i.v. injection of 52.8 nM (**A**) HB2-Sap 2-mer IT (— —), (**B**) HB2-Sap 1-mer IT (- - -), (**C**) HB2 antibody plus saporin at a 2-mer equivalent ratio (···), (**D**) HB2 antibody plus saporin at a 1-mer equivalent ratio (----) or (**E**) PBS sham treated (—)

synthesis inhibition to half that seen in untreated controls ($IC_{50}$), the 2-mer IT required 3.7 times more antibody (1.1 × $10^{-8}$ M) than the 1-mer IT (3 × $10^{-9}$ M) to achieve the same effect (Figure 6). Total blockade of both 1-mer IT-and 2-mer IT-mediated cytotoxicity was only achieved at a gross excess antibody concentration of $10^{-7}$ M. Each IT was present in the assay system at a fixed concentration of 1 × $10^{-10}$ M, and we can therefore conclude that a 1000-fold molar excess of native HB2 antibody is required to completely block IT cytotoxicity. Native HB2 antibody alone, used over the same concentration range but without IT, had no effect on protein synthesis levels (Figure 6).

### Effects of HB2-Sap 1-mer and 2-mer ITs on HSB-2 cell proliferation

The effects of four different equimolar concentrations of 1-mer or 2-mer IT on HSB-2 cell proliferation in vitro are shown in Figure 7. The 2-mer IT was more effective at inhibiting HSB-2 cell proliferation than the 1-mer IT, delaying cell growth by several days. Used over the same concentration range, neither I T had any effect on the growth of the $CD7^-$ cell line NALM-6 (data not shown).

### Pharmacokinetic profiles of 1-mer and 2-mer immunotoxins

A two-compartment first-order pharmacokinetic model was used to fit the 1-mer and 2-mer IT serum concentration data. The pharmacokinetic parameters obtained for both the 1-mer and 2-mer ITs in HSB-2 tumour-bearing SCID mice are shown in Table 2. It was apparent that there was a very marked increase in the $t_{1/2}$ (α) and $t_{1/2}$ (β) phases for the 2-IT and a resultant larger AUC for the 2-mer IT.

### Toxicity of 1-mer and 2-mer ITs in SCID mice

All SCID mice receiving a single, double or triple i.v. 10-μg dose (52.8 nM) of 1-mer IT given on alternate days survived with no apparent toxicological problems. In contrast, some animals receiving molar equivalent doses of 2-mer IT encountered significant toxicological problems. All animals receiving a single 11.58-μg dose (52.8 nM) of 2-mer IT survived, while only 80% and 50% of animals receiving two and three doses, respectively, survived (Figure 8). Post mortem and histopathological examination of animals receiving 2-mer IT that died intercurrently revealed varying degrees of liver parenchymal cell necrosis and some renal tubular necrosis; such lesions have been seen with saporin ITs in mice previously (DJ Flavell, unpublished observations).

**Table 3** Mean survival times of groups of HSB-2-challenged SCID mice receiving therapy with 1-mer or 2-mer HB2-Sap immunotoxins, HB2 antibody plus saporin at a 1-mer or 2-mer molar equivalent ratio or PBS sham treated

| Group | Therapy | Mean survival (days) | Survivors at 295 days (%) |
|---|---|---|---|
| A | HB2-Sap 2-mer IT | 136.9 | 70 |
| B | HB2-Sap 1-mer IT | 123.3 | 60 |
| C | HB2-sap Ab plus saporin (2-mer equivalent ratio) | 88.7 | 0 |
| D | HB2 Ab plus saporin (1-mer equivalent ratio) | 83.8 | 10 |
| E | PBS sham treated | 59.7 | 0 |

### Therapeutic efficacy of HB2-Sap 1-mer and 2-mer immunotoxins in HSB-2 leukaemia-bearing SCID mice

Survival curves obtained for groups of SCID HSB-2 mice treated with a single 52.8-nM i.v. dose of 1-mer or 2-mer HB2-Sap ITs, unconjugated HB2 antibody plus saporin or PBS sham treated are shown in Figure 9, and the data is summarized in Table 3. The presence of HSB-2 tumour growth was confirmed grossly and by immunocytochemistry in all animals dying intercurrently during the course of the study. There were no survivors in the PBS sham-treated control group, all animals dying with disseminated leukaemia by 92 days with a mean survival of 59.7 days. Animal groups treated with 1-mer or 2-mer IT had mean survival times of 123.3 and 136.9 days, respectively, with a respective 60% and 70% of animals alive and disease-free in these groups on day 295 at the termination of the experiment. Treatment with native HB2 antibody plus saporin at two different ratios equivalent in molar terms to 1-mer and 2-mer IT did have a therapeutic effect, increasing mean survival to 83.8 and 88.7 days, respectively, with only one animal from these groups surviving disease-free for the duration of the study. Log-rank analysis revealed that the differences between the PBS sham-treated group and 1-mer IT-and 2-mer IT-treated groups were highly significant ($P$=0.0316 and $P$=0.0071 respectively). However, the difference between the 1-mer and 2-mer IT therapy groups was not significant ($P$=0.6306). The improved survival seen when a mixture of native HB2 antibody plus saporin was used at an equivalent 1-mer (but not 2-mer) molar ratio was just significant when compared with sham-treated controls ($P$=0.0573).

### DISCUSSION

This work was undertaken to determine whether an anti-CD7-saporin IT, HB2-Sap 2-mer, containing two saporin molecules per antibody molecule performed better therapeutically in vivo against the human $CD7^+$ T-ALL cell line HSB-2 than HB2-SAP 1-mer IT containing just one saporin molecule. We have clearly shown that the 2-mer IT was more than five times more effective in vitro at inhibiting protein synthesis in target HSB-2 cells than the 1-mer IT

© Cancer Research Campaign 1997

and that a proportionately greater excess of native HB2 antibody was required to block the cytotoxic activity of the 2-mer than was required for the 1-mer IT. Similarly, other workers have described similar increases in in vitro potency of ITs containing two toxin molecules per unit antibody (Marsh and Neville, 1986; Ghetie et al, 1993). In the present study, it was also clearly demonstrated that the 2-mer IT inhibited in vitro HSB-2 cell proliferation more effectively than the 1-mer IT. This improved in vitro performance of the 2-mer IT occurred despite an apparent reduced binding capacity of 2-mer for HSB-2 cells as demonstrated by flow cytometry. The flow cytometry data indicate that the reduced binding capacity of the 2-mer IT is probably due to steric hindrance of antigen binding by the second saporin moiety in a proportion of 2-mer immunotoxin molecules. This contention is further supported by the observation that, in a competitive inhibition study, the 1-mer IT had a higher binding affinity for CD7 than the 2-mer IT. The apparent disparity that exists between the reduced binding capacity of 2-mer IT and yet the apparent increased in vitro effectiveness over 1-mer IT at inhibiting both protein synthesis and cell proliferation in $CD7^+$ target cells may possibly be explained by an improved internalization of the 2-mer IT to the appropriate intracellular compartment. Perhaps, more simply, the improved in vitro efficacy of the 2-mer IT may be an entirely numerical effect, with the overall number of saporin molecules delivered per unit CD7 target molecule ovecompensating for the reduced binding capacity of this IT. In this respect, the relative high efficiency with which the CD7 molecule is able to deliver saporin to the appropriate cellular compartment is underscored by the very large 1000-fold excess of native HB2 antibody that is required to block both 1-mer and 2-mer IT cytotoxicity in vitro. These data show that the relatively small amount (12%) of contaminating native HB2 antibody present in the 1-mer preparation therefore has an insignificant effect on IT performance; and moreover our results indicate that the reduced binding capacity of the 2-mer IT may be insufficient to diminish the beneficial effects of delivering two saporin molecules per unit CD7 target molecule. Such issues would likely be resolved by quantifying the receptor-mediated endocytosis rates for both the 1-mer and 2-mer ITs and by relating these to the number of immunotoxin molecules bound at the cell surface. Despite the improved in vitro performance of the 2-mer IT, the therapeutic performance of 1-mer IT and 2-mer IT in the SCID mouse model of disseminated HSB-2 leukaemia was almost identical, with the 2-mer IT performing marginally though not significantly better than the 1-mer IT.

Several workers have described the in vivo therapeutic efficacy of anti-CD7 ITs against human T-cell malignancies (Fishwild et al, 1992; Jansen et al, 1992; Flavell et al, 1995*a*; Morland et al, 1994), although all these studies have used immunotoxins composed of a mixture of ITs containing varied toxin – antibody ratios. Similar to the present study, these studies have also demonstrated the in vivo anti-tumour effects of native unconjugated anti-CD7 antibody, which although pronounced are less significant than the therapeutic effects obtained when anti-CD7 ITs are used for therapy. It is assumed that SCID effector cells, particularly NK cells that are still present in SCID mice (Dorshkind et al, 1985), are responsible for this anti-tumour effect directed against antibody-coated tumour cells, and we have recently made findings that strongly support this contention (DJ Flavell et al, in preparation). Thus, it is true to say that the in vivo anti-tumour effects seen in SCID mouse and similar animal models of human T-ALL with anti-CD7 ITs in reality represent a response to two different cytotoxic mechanisms. In order to determine the differential contribution that such diverse cytotoxic mechanisms contribute to the eventual therapeutic outcome, we are currently undertaking studies in NOD-SCID mice, which are naturally deficient in NK cells.

The present study addressed the question of both in vitro and in vivo immunotoxin potency in the context of the number of toxin molecules carried by a single antibody molecule to a single antigen unit on the target cell surface. The improved in vitro performance of the 2-mer IT over the 1-mer IT, despite the reduced binding capacity of the 2-mer IT, is probably due to the delivery of twice the number of saporin molecules per unit target antigen and their resultant internalization by receptor-mediated endocytosis to the appropriate intracellular compartment, as discussed earlier. In addition to the 2-mer IT showing a demonstrably improved $IC_{50}$ over the 1-mer IT, kinetic studies also revealed that the 2-mer IT inactivated protein synthesis in target HSB-2 cells more rapidly than the 1-mer IT. We have described a similar phenomenon previously with a combination of two bispecific antibodies delivering saporin to HSB-2 cells via CD7 and CD38 target molecules, resulting in a more rapid inactivation of protein synthesis (Flavell et al, 1992). Although unproven, the most likely explanation for this would seem to be that increasing the overall number of toxin molecules delivered to and subsequently internalized by the target cell would increase the resultant probability of achieving a ‘hit’ on the target ribosome.

The finding that the increased in vitro potency of 2-mer IT over 1-mer IT did not translate into an improved in vivo therapeutic outcome in the SCID mouse model was disappointing. The 2-mer IT was considerably more toxic in the SCID mouse than the 1-mer IT, an observation that may possibly be explained by the greater serum half-life of 2-mer IT. Myers et al (1995) have made similar observations with an anti-CD19 immunotoxin (B43-PAP) in a SCID mouse model of human cALL and were also able in this instance to demonstrate that the IT species containing two PAP molecules was significantly more effective therapeutically than the single toxin-containing species. Ghetie et al (1995) undertook studies based upon one of our earlier reports (Flavell et al, 1995*b*) and showed that an anti-CD19 immunotoxin containing two ricin A chain toxin molecules per antibody molecule was more potent at inactivating protein synthesis in target cell lines in vitro than an equivalent IT containing just a single toxin molecule. However, there was little difference between the therapeutic performance of the one and two ricin A chain-containing ITs in a SCID–Daudi mouse model of human lymphoma. An anti-CD25 IT containing one or two ricin A-chain molecules performed almost identically both in vitro and in vivo. In contrast, an anti-CD22 IT containing two ricin A chain molecules per antibody molecule performed significantly better than an equivalent single toxin containing IT not only in vitro but also in vivo in the same SCID–Daudi model. In sharp contrast to our own findings, these workers report that all three of their two toxin-containing ITs were no more toxic in the mouse than the one toxin-containing ITs. The reasons for this are not clear, but it may reflect the greater sensitivity of the mouse to the hepatic and renal toxicity of saporin.

It seems entirely possible that the properties and nature of the target molecule dictate the therapeutic potency of a given IT directed against this target. However, this alone cannot account for the disparities that appear to exist between in vitro and in vivo IT performance. Other issues, such as IT clearance rates from the blood, accessibility of target cells by the IT and heterogeneity of target antigen expression (Flavell et al, 1995*a*) by the target cells,

© Cancer Research Campaign 1997

are equally important factors that may have a profound influence on in vivo therapeutic efficacy. In the present study, the lack of improvement may relate to in vivo tumour accessibility, which may be reduced for the 2-mer IT because of its larger molecular size. The 2-mer IT, although more than 5 times more potent in vitro, also has a demonstrably lower in vivo therapeutic index because of its increased toxicity in the SCID mouse. While it was possible to administer three 10-μg doses of the 1-mer IT to SCID mice without any attendant toxicity, we were only able to give a single 10-μg dose of the 2-mer IT. This increased toxicity is almost certainly due to the increased serum half-life of the 2-mer IT and the resultant larger AUC. Ghetie et al (1995), who did not find any increased toxicity in their two ricin A chain-containing ITs, did not provide any pharmacokinetic data on these ITs, and it is therefore not possible to comment on possible reasons for this in relation to our own findings. We thus conclude that there would appear to be no therapeutic advantage to be gained, at least in the presently described SCID model of human T-ALL, in constructing ITs with more than one saporin molecule linked to a single antibody molecule; indeed, the increased toxicity that is observed is a major disadvantage. However, the results of others suggest that different target molecules and different antibodies directed against the same target molecule may behave differently. To this end, we are currently exploring the in vivo therapeutic prformance of other 1-mer and 2-mer ITs constructed with different anti-CD7, -CD19, -CD22 and -CD38 antibodies, each with different epitope specificities.

### ACKNOWLEDGEMENTS

This work was supported by grants from The Leukaemia Research Fund and Leukaemia Busters (children's leukaemia research). We would like to thank Dr Herbie Newell for assistance with pharmacokinetic calculations and L Emery for technical assistance.

### REFERENCES

Adams RA, Pothier L, Flowers A, Lazarus H, Farber S and Foley GE (1970) The question of stemlines in human acute leukemia. Comparison of cells isolated *in vitro* and *in vivo* from a patient with acute lymphoblastic leukemia. *Exp Cell Res* **62**: 5–10

Amlot PL, Stone MJ, Cunningham D, Fay J, Newman J, Collins R, May RM, Carthy M, Richardson J, Ghetie V, Ramilo O, Thorpe, PE, Whr JW and Vitetta ES (1993) A phase I study of an anti-CD22-deglycosylated ricin A chain immunotoxin in the treatment of B-cell lymphomas resistant to conventional therapy. *Blood* **82**: 2624–2633

Dorshkind K, Pollack SB, Bosma MJ and Phillips RA (1985) A Natural killer cells are present in mice with severe combined immunodeficiency. *J Immunol* **134**: 3798–3801

Falini B, Bolognesi A, Flenghi L, Tazzari PL, Broe MK, Stein H, Durkop H, Aversa F, Cornell P, Pizzolo G, Barbabietola G, Sabattini E, Pileri S, Matelli MF and Stirpe F (1992) Response of refractory Hodgkin's disease to monoclonal anti-CD30 immunotoxin. *Lancet* **339**: 1195–1196

Fishwild DM, Aberle S, Bernhard SL and Kung AHC (1992) Efficacy of an anti-CD7-Ricin A chain immunoconjugate in a novel murine model of human T-Cell leukaemia. *Cancer Res* **52**: 3056–3062

Flavell DJ, Cooper S, Morland B and Flavell SU (1991) Characteristics and performance of a bispecific $F(ab'g)_2$ antibody for delivering saporin to a $CD7^+$ human acute T-cell leukaemia cell line. *Br J Cancer* **64**: 274–280

Flavell DJ, Cooper S, Morland B, French R and Flavell SU (1992) Effectiveness of combinations of bispecific antibodies for delivering saporin to human acute T-cell lymphoblastic leukaemia cell lines via CD7 and CD38 as cellular target molecules. *Br J Cancer* **65**: 545–551

Flavell DJ, Boehm DA, Okayama K, Kohler JA and Flavell SU (1994) Therapy of human T-cell acute lymphoblastic leukaemia in severe combined immunodeficient mice with two different anti-CD7-saporin immunotoxins containing hindered or non-hindered disulphide cross linkers. *Int J Cancer* **58**: 407–414

Flavell DJ, Boehm D, Emery L, Noss A and Flavell SU (1995*a*) Therapy of human B-cell lymphoma bearing SCID mice is more effective with anti-CD19 and anti-CD38 saporin immunotoxins used in combination than with either immunotoxin used alone. *Int J Cancer* **62**: 337–344

Flavell DJ, Boehm DA, Emery L, Noss A and Flavell SU (1995*b*) Comparison of the therapeutic efficacy of anti-CD7 saporin immunotoxins constructed with one or two saporin moieties per immunotoxin molecule in SCID mice with human T-cell leukaemia. *4th International Symposium on Immunotoxins*, pp. 135, Myrtle Beach, SC

Ghetie V, Swindell E, Uhr JW and Vitetta ES (1993) Purification and properties of immunotoxins containing one vs. two deglycosylated ircin A chains. *J Immunol Method* **166**: 117–122

Ghetie V, Engert A, Schnell R and Vitetta ES (1995) The in vivo anti-tumour activity of immunotoxins containing two versus one deglycosylated ricin A chains. *Cancer Let* **98**: 97–101

Grossbard ML, Lambert JM, Goldmacher VS, Spector NL, Kinsella J, Eliseo L, Coral F, Taylor JA, Blattler WA, Epstein CL and Nadler LM Anti-B4-blocked ricin: a phase I trial of 7-day continuous infusion in patients with B-cell neoplasms. *J Clin Oncol* **11**: 726–737

Jansen B, Vallera DA, Jaszcz WB, Nguyen D and Kersey JH (1992) Successful treatment of human acute T-cell leukaemia in SCID mice using the anti-CD7-deglycosylated ricin A chain immunotoxin DA7. *Cancer Res* **52**: 1314–1321

Laemmli UK (1970) Cleavage of structural proteins during the assembly of the head of bacteriophage T4. *Nature* **227**: 680

Lambert JM, Senter PD, Yau-Young A, Blatter WA and Goldmacher VS (1983) Purified immunotoxins that are reactive with human lymphoid cells. MAbs conjugated to the RIP's gelomin and PAP. *J Biol Chem* **260**: 12035–12041

Marsh JW and Neville DM (1986) Kinetic comparison of ricin immunotoxins: biricin conjugate has potentiated cytotoxicity. *Biochemistry* **25**: 4461–4467

May RD, Wheeler HT, Finkelman FD, Uhr JW and Vitetta ES (1991) Intracellular routing rather than cross linking or rate of internalization determines the potency of immunotoxins directed against different epitopes of sIgD on murine B-cells. *Cell Immunol* **135**: 490–500

Morland BJ, Barley J, Boehm D, Flavell SU, Ghaleb N, Kohler JA, Okayama K, Wilkins B and Flavell DJ (1994) Effectiveness of HB2 (anti-CD7)-saporin immunotoxin in an *in vivo* model of human T-cell leukaemia developed in severe combined immunodeficient mice. *Br J Cancer* **69**: 279–285

Myers DE, Yanishevski Y, Masson E, Irvin JD, Evans WE and Uckun FM (1995). Favorable pharmacodynamic features and superior anti-leukemic activity of B43 (anti-CD19) immunotoxins containing two Pokeweed antiviral protein molecules covalently linked to each monoclonal antibody molecule. *Leukemia Lymphoma* **18**: 93–102

Preijers FWMB (1988) Relationship between internalisation and cytotoxicity of ricin A chain immunotoxins. *Br J Haematol* **70**: 289–294

Press OW, Martin PJ, Thorpe PE and Vitetta ES (1988) Ricin A chain containing immunotoxins directed against different epitopes on the CD2 molecule differ in their ability to kill normal and maignant T-cells. *J Immunol* **141**: 4410–4417

Stirpe F, Gasperi-Campani A, Barbieri L, Falasca A, Abbondanza A and Stevens WA (1983) Ribosome-inactivating proteins from the seeds of *Saponaria Officinalis* L. (soapwort), of *Agrostemma Githago* L. (corn cockle) and of *Asparagus Officinalis* L. (asparagus), and from the latex of *Hura Crepitans* L. (sandbox tree). *J. Biochem*, **216**: 617–625

Thorpe PE, Brown ANF, Jr JAGB, Foxwell BMJ and Stirpe F (1985) An immunotoxin composed of monoclonal anti-Thy 1.1 antibody and a ribosome-inactivating protein from *Saponaria Officinalis:* potent antitumor effects in vitro and in vivo. *J Natl Cancer Inst* **75**: 151–159

Van-Oosterhout YVJM, Van-Den-Herrik-Oudijk IE, Wessels HMC, De-Witte T, Van-de-Winkel JGJ and Preijers FWMB, (1994) Effect of isotype on internalization and cytotoxicity of CD19-ricin A immunotoxins. *Cancer Res* **54**: 3527–3532

Vitetta ES, Stone M, Amlot P, Fay J, May R, Till M, Newman J, Clark P, Collins R, Cunningham D, Ghetie V, Uhr JW and Thorpe PE (1991) Phase I immunotoxin trial in patients with B-cell lymphoma. *Cancer Res* **51**: 4052–4058

Wargalla UC and Reisfeld RA (1989) Rate of internalization of an immunotoxin correlates with cytotoxic activity against human tumor cells. *Proc Natl Acad Sci USA* **86**: 5146–5150

© Cancer Research Campaign 1997